# 

**Published:** 1858-04

**Authors:** 


Vol. 1, APRIL, 1858, No. 1.

### THE DENTAL REPORTER:

AN INDEPENDENT JOURNAL,

DEVOTED TO

**Dental Progress and Improvements,**

IN THE

**MANUFACTURE AND USE OF**

INSTRUMENTS AND MATERIALS.

EDITED BY JNO. T. TOLAND.

Published Quarterly by

**JNO. T. TOLAND,**

MANUFACTURER OF

DENTAL INSTRUMENTS & WHOLESALE DEALER IN DENTISTS' MATERIALS.

**38 West Fourth Street, Cincinnati.**

*PRICE,* **25** *CENTS A YEAR IN* **ADVANCE,** *SINGLE COPIES* **10** *CENTS.*

